# Efficient 2D-DOA Estimation Based on Triple Attention Mechanism for L-Shaped Array

**DOI:** 10.3390/s25082359

**Published:** 2025-04-08

**Authors:** Yonghong Zhao, Xiumei Fan, Jisong Liu, Yuxing Li, Lyulong Yao, Junlong Wang

**Affiliations:** 1 School of Automation and Information Engineering, Xi’an University of Technology, Xi’an 710048, China; zhaoyh2018@xaut.edu.cn (Y.Z.); 2230320112@stu.xaut.edu.cn (J.L.); liyuxing@xaut.edu.cn (Y.L.); 2240320156@stu.xaut.edu.cn (L.Y.); 2230321285@stu.xaut.edu.cn (J.W.); 2 Shaanxi Key Laboratory of Complex System Control and Intelligent Information Processing, Xi’an University of Technology, Xi’an 710048, China

**Keywords:** direction-of-arrival estimation, L-shaped array, deep learning, triple attention mechanism

## Abstract

Accurate direction-of-arrival (DOA) estimation is crucial to a variety of applications, including wireless communications, radar systems, and sensor arrays. In this work, we propose a novel deep convolutional neural network (DCN) called TADCN for 2D-DOA estimation using an L-shaped array. The network achieves high estimation performance through a triple attention mechanism (TAM). Specifically, the new architecture enables the network to capture the relationships across the channel, height, and width dimensions of the signal sample features, thereby enhancing the feature extraction capability and improving the resulting spatial spectrum. To this end, the spatial spectrum is processed by the proposed spectrum analyzer to yield high-precision DOA estimation results. An automatic angle matching method based on TADCN is employed for estimating the pairing between the estimated azimuth and elevation DOA sets. Furthermore, the overall efficiency is enhanced through the parallel processing of the angle estimation and matching networks. Simulation results demonstrate that the proposed algorithm outperforms traditional methods and deep learning-based approaches for various noise levels and snapshots while maintaining better estimation performance even in the presence of correlated signal sources.

## 1. Introduction

Direction-of-arrival (DOA) estimation has always been an active research area, playing an essential role in numerous applications, such as radar, sonar, and wireless communications [1,2,3,4,5,6]. An important goal of DOA estimation is to accurately locate closely spaced sources in the presence of a low signal-to-noise ratio (SNR), a limited number of available snapshots, and correlations between signals [3,7,8,9]. A typical model-based method for DOA estimation is multiple signal classification (MUSIC) [10,11]. MUSIC leverages the orthogonality between the signal subspace and the noise subspace, and angle estimation is derived by identifying the peaks of the MUSIC pseudo-spectrum evaluated over a predefined grid. As the grid becomes more densely divided, the computational cost grows, demanding significant resources and hindering real-time processing capabilities. The estimation performance significantly degrades under low-SNR conditions or in the presence of highly correlated signals. To overcome these limitations, researchers have developed various enhanced versions of the MUSIC algorithm [8,12,13,14], such as the Root-MUSIC algorithm. Another typical algorithm is estimation of signal parameters via rotational invariance techniques (ESPRIT). ESPRIT gives the final estimation of signal parameters through eigenvalue decomposition rather than spectral search, making it more computationally efficient than MUSIC [15,16,17,18]. Besides MUSIC and ESPRIT, there are several other traditional model-based DOA estimation methods [2,5,19,20,21,22]. The Sparse Iterative Covariance-based Estimation (SPICE) method, proposed by P. Stoica et al., is a technique based on sparse estimation and covariance matrix fitting. It improves estimation accuracy through an iterative optimization process, without relying on traditional methods such as grid search or eigenvalue decomposition (EVD). Unlike other sparse estimation methods, SPICE does not require the manual selection of hyperparameters, thus avoiding the complex selection process in traditional approaches. Although SPICE performs well in low-SNR environments, it may still face challenges when dealing with closely spaced signal sources [22]. The aforementioned methods require the establishment of rigorous mathematical models. When the models do not match, such as array error, the estimation performance will be severely degraded.

Furthermore, the choice of the array structure plays a crucial role in obtaining superior performance. Investigations into the impact of different array geometries on DOA estimation performance have shown that planar arrays provide significantly higher elevation angle estimation accuracy than volumetric arrays [23]. Planar arrays typically include linear arrays, rectangular arrays, circular arrays, etc. [2,19,24,25,26,27]. In addressing 1D-DOA estimation tasks, the linear array is a frequently used array configuration due to its relatively low computational complexity, making it highly efficient for the experimental testing of various methods [2]. For 2D-DOA estimation, rectangular arrays offer higher resolution but require greater computational resources and processing power [28]. The authors in [29] employed higher-order unitary propagation operators to enhance the spatial sampling accuracy of the signals and effectively avoid the high computational overhead typically associated with traditional methods. For the uniform circular array (UCA), a method based on fourth-order cumulants and beamspace transformation has been proposed in [30], which can obtain a larger virtual array aperture to enhance the robustness of the algorithm in low-SNR environments and a limited number of snapshots. In addition, M. Boddi et al. [31] used an iterative method to estimate the DOAs of plane wave signals received by a UCA. This method not only considers mutual coupling effects modeled as gain and phase errors but also transforms the data collected by the UCA into a virtual uniform linear array to enhance the accuracy of DOA estimation. Besides the aforementioned typical arrays, there are also non-circular arrays, such as the L-shaped array used in this study [28,29,32,33,34]. Non-circular arrays, with more flexible geometric configurations, account for the irregular spatial arrangement of array elements, which results in better spatial resolution [33]. The L-shaped array, with its simple and efficient structural characteristics, has been widely applied in various 2D-DOA estimation problems [1,35,36,37]. Y. Yang et al. [1] used an inclined projection operator to fill the gaps in the cross-difference co-array of the nested L-shaped array and reconstruct the virtual correlation matrix, which is then used to estimate the 2D-DOA. This operation significantly improves the degrees of freedom and resolution of DOA estimation under underdetermined conditions. G. Lu et al. [37] used projection approximation subspace tracking to dynamically update the signal subspace and solve the real-time 2D-DOA estimation problem for the L-shaped array by using the ESPRIT algorithm. The algorithm avoids the eigenvalue decomposition of the covariance matrix, thereby significantly reducing computational complexity. The aforementioned studies indicate that the L-shaped array can decompose the 2D-DOA estimation problem into two 1D problems. This decomposition strategy not only enhances estimation performance but also reduces the demand for system resources, making the L-shaped array highly robust in resource-constrained scenarios. Nevertheless, the limitations of these estimation algorithms still pose a risk of error accumulation in practical applications, especially in scenarios with high noise levels or strongly correlated signal sources.

In recent years, machine learning theory has been an active research area and has been applied to various fields. For the problem of DOA estimation, it can be used to learn the complex nonlinear relationship between array data and signal direction by constructing a labeled DOA training dataset. Once the signal features are extracted based on the underlying signal model, no predefined mathematical model is needed for the subsequent estimation process. Moreover, network parameters are automatically optimized during training by using algorithms like Adam. Earlier machine learning methods with high computational efficiency, such as radial basis function and support vector regression, are sensitive to data outliers and rely heavily on the distribution of the training data. Consequently, these methods may struggle to maintain optimal performance in real-world applications, especially when the input data deviate from the assumed distribution or contain significant noise [38,39,40,41,42,43,44]. Subsequently, researchers have combined the advantages of deep learning and traditional machine learning techniques by designing more efficient and robust network architectures to further improve the accuracy of DOA estimation, such as deep neural network (DNN), convolutional neural network (CNN), and residual–deep convolutional neural network (RDCN) [45]. These methods maintain good performance even under conditions of limited snapshots and low SNR [25,46,47]. However, deep learning-based methods for DOA estimation still face several challenges, including sensitivity to low SNR and multipath interference, reliance on large volumes of labeled data, limited generalization to varying array configurations, and vulnerability to overfitting in noisy or dynamic signal environments.

To address these issues, recent studies have proposed various strategies from both model design and input representation perspectives. Hassan et al. [48] introduced a CNN-based approach that directly regresses DOAs from the covariance matrix, demonstrating strong performance under multipath and low-SNR conditions. Similarly, Liu et al. [49] developed a non-deep learning method based on iterative optimization with semi-unitary constraints, effectively handling multipath association without requiring prior knowledge of the propagation environment. In parallel, other studies have widely adopted the covariance matrix as network input, as it captures second-order statistical information and improves robustness under noise. Tan et al. [50] proposed a complex-valued CNN with phasor normalization (C-LeDIM-net) which enhances estimation accuracy by explicitly preserving phase features in the input covariance structure. Zhao et al. [51] designed a deep CNN that maps the covariance matrix into a binary angular domain, significantly improving estimation under conditions of limited snapshots and low SNR. In recent work, several representative methods have been proposed. Lu et al. [52] proposed a CNN-based improved CAPON algorithm that preserves the blind estimation advantage of Minimum Variance Distortionless Response (MVDR) while effectively reducing estimation error through cyclic noise reduction. Mylonakis et al. [53] introduced a spatial attention-enhanced CNN combined with transfer learning, achieving robust performance across varying array configurations with minimal retraining effort. These approaches significantly enhance DOA estimation performance in complex scenarios, promoting greater efficiency and broader applicability in real-world deployments.

In this paper, we propose a fusion framework that introduces triple attention mechanisms into deep convolution for 2D-DOA estimation in coherent multipath scenarios. First, we design the input data based on the L-shaped array for the proposed network model, called Triple Attention Deep Convolution Network (TADCN). Subsequently, the proposed TADCN model is used to estimate the DOAs and generate the corresponding angle pairings. Finally, an angle output module is constructed to achieve more accurate 2D-DOA estimation results. The contributions of our work are summarized as follows:

- A completely new model, called TADCN, is proposed; it combines deep convolutional neural network (DCN) and the triple attention mechanism (TAM). The TADCN model implements end-to-end optimization, which can automatically adjust its parameters during training. Unlike traditional attention mechanisms, which fail to capture the multi-dimensional dependencies of signals, TADCN can be used to extract valuable spatial features from the raw data. Experimental results show that TADCN demonstrates superior robustness to noise.
- We design a new architecture for 2D-DOA estimation by using the L-shaped array configuration. This structure is capable of simultaneously estimating the angle information and achieving automatic pairing. The angle estimation network is used to estimate the azimuth and elevation angles of the signals based on the covariance matrix. The angle matching network is used to pair the estimated azimuth and elevation angles to form the final angle combination. It also demonstrates strong identification capabilities in multipath propagation and multi-source scenarios. Compared with traditional model-based DOA estimation methods, the proposed architecture can automatically learn signal features without relying on extensive prior knowledge. This allows the method to avoid the dependence on complex mathematical models that traditional methods often require.
- The performance of the proposed solution is verified over an extensive set of simulated experiments and compared against traditional MUSIC and ESPRIT algorithms, as well as other deep learning methods, in various experimental setups. The experimental results show that the proposed method has superior performance.

The remainder of this paper is organized as follows: Section 2 introduces the signal model based on the L-shaped array and discusses the signal information contained in the covariance matrix. Section 3 presents the architecture of the proposed model in detail. Section 4 introduces the construction and training process of the angle estimation network and the angle matching network. The advantages and disadvantages of the proposed framework are explored and compared with other common methods by using simulated experiments in Section 5. Finally, Section 6 concludes the paper.

## 2. Singal Model

**Figure 1:**
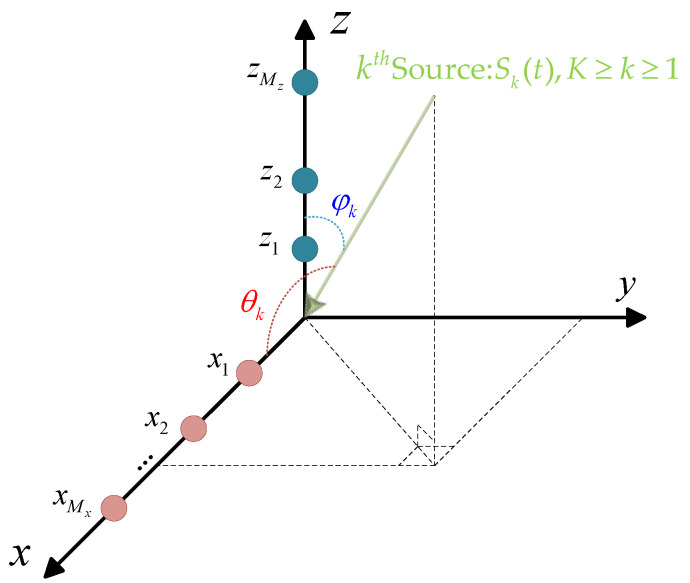
L-shaped array geometry.

Let us consider an L-shaped array composed of two linear orthogonal arrays, with $M_{x}$ and $M_{z}$ elements located at positions ${\left\{x_{m}^{\,}\right\}}_{m=1}^{M_{x}}$ and ${\left\{z_{m}^{\,}\right\}}_{m=1}^{M_{z}}$, respectively, as shown in Figure 1. We further assume that K narrowband far-field sources are in the surveillance region, impinging on the L-shaped array from $\left(\theta_{k},\varphi_{k}\right)$, k=1,…,K, where $\theta_{k}$ represents the angle between the incident direction and the x-axis, while $\varphi_{k}$ is the angle relative to the z-axis. Then, the received signals on the L-shaped array in the x-axis and z-axis subarrays are obtained as

(1) $$\begin{array}{l} y_{x}(t)=A_{x}\left(\theta \right)s(t)+n_{x}(t)\\ y_{z}(t)=A_{z}\left(\varphi \right)s(t)+n_{z}(t) \end{array},$$

where $s(t)={\left[s_{1}(t),s_{2}(t),\ldots ,s_{K}(t)\right]}^{T}\in {\mathbb{C}}^{K}$ denotes the random signal received vector. $n_{x}(t)\in {\mathbb{C}}^{M_{x}\times 1}$ and $n_{z}(t)\in {\mathbb{C}}^{M_{z}\times 1}$ represent the vectors of additive noise of the x-axis and z-axis subarrays, which are temporally and spatially white complex Gaussian with zero mean and variance $\sigma_{n}^{2}$ and are uncorrelated with incident signals. The matrices $A_{x}\left(\theta \right)$ and $A_{z}\left(\varphi \right)$ are the so-called array manifold matrices, whose kth columns contain the angle information from the kth source (at location $\theta_{k}$, $\varphi_{k}$) to the x-axis and z-axis, respectively. Columns $a_{x}(\theta_{k})$ of $A_{x}\left(\theta \right)$, for $k\in \left\{1,\cdots ,K\right\}$, are called steering vectors. We similarly define $a_{z}(\theta_{k})$ of $A_{z}\left(\theta \right)$. The steering vectors of the kth source for the x-axis and z-axis subarrays can be represented by

(2) $$\begin{array}{l} a_{x}(\theta_{k})={\left[\exp(j2\pi x_{1}\cos(\theta_{k})/\lambda ),\;\ldots ,\;\exp(j2\pi x_{M_{x}}\cos(\theta_{k})/\lambda )\right]}^{T}\in {\mathbb{C}}^{M_{x}\times 1}\\ a_{z}(\varphi_{k})={\left[\exp(j2\pi z_{1}\cos(\varphi_{k})/\lambda ),\;\ldots ,\;\exp(j2\pi z_{M_{z}}\cos(\varphi_{k})/\lambda )\right]}^{T}\in {\mathbb{C}}^{M_{z}\times 1} \end{array}$$

where λ is the signal wavelength and ${\left(\cdot \right)}^{T}$ denotes the transpose operation.

From (1), we can see that $\theta_{k}$ is only included in the received data of the x-axis subarray, while $\varphi_{k}$ is only included in the received data of the z-axis subarray. So, this representation allows us to exchange the problem of two-dimensional DOA estimation for the problem of two one-dimensional linear array estimations. In addition, it can be seen that $y_{x}(t)$ and $y_{z}(t)$ have the same structure. To simplify the analysis, we consider only the x-axis subarray for the remainder of this section. In order to improve the performance of DOA estimation, especially at low SNR, we consider the statistics of the received signal. For the x-axis subarray, the covariance matrix $R_{xx}$ is defined as the second-order statistic of the received signal $y_{x}(t)$:

(3) $$R_{xx}=E[y_{x}(t)y_{x}^{H}(t)]=A_{x}\left(\theta \right)R_{s}A_{x}^{H}\left(\theta \right)+E\left[n_{x}(t)n_{x}^{H}(t)\right]$$

where ${\left(\cdot \right)}^{H}$ is the conjugate transpose operation and $R_{s}=E[s(t)s^{H}(t)]$ is the covariance matrix of the signal sources. According to the description of noise in (1), the noise covariance matrix is given by

(4) $$E[n_{x}(t)n_{x}^{H}(t)]=\sigma_{n}^{2}I_{M_{x}}$$

Therefore, the covariance matrix of the received signal $R_{xx}$ can be expressed as

(5) $$R_{xx}=A_{x}R_{s}A_{x}^{H}+\sigma_{n}^{2}I_{M_{x}}$$

In real applications, the ideal covariance matrix is not available, which is estimated by the sample covariance matrix with a finite number of snapshots. We can obtain the estimation of $R_{xx}$ as follows:

(6) $${\hat{R}}_{xx}=\frac{1}{L}\sum_{t=1}^{L}y_{x}(t)y_{x}^{H}(t)$$

where the estimated values for quantities are expressed as $\hat{\left(\cdot \right)}$ and L is the number of snapshots. Similarly, the estimation of the covariance matrix for the z-axis subarray can be calculated as

(7) $${\hat{R}}_{zz}=\frac{1}{L}\sum_{t=1}^{L}y_{z}(t)y_{z}^{H}(t)$$

Except for the mappings ${\hat{R}}_{xx}\to \theta_{k}$ and ${\hat{R}}_{zz}\to \varphi_{k}$, there is another very important issue, which is the angular pairing, that needs to be addressed to obtain the knowledge of the location of the sources. Here, we introduce the cross-covariance matrix $R_{xz}$, which describes the correlation between the signals of the x-axis and z-axis subarrays, defined as follows:

(8) $$R_{xz}=E[x(t)z^{H}(t)]=A_{x}(\theta )R_{s}A_{z}^{H}(\varphi )$$

$R_{xz}$ is used to match $\theta_{k}$ and $\varphi_{k}$ to complete two-dimensional DOA estimation. In practice, $R_{xz}$ is estimated as

(9) $${\hat{R}}_{xz}=\frac{1}{L}\sum_{t=1}^{L}y_{x}(t)y_{z}^{H}(t)$$

**Figure 2:**
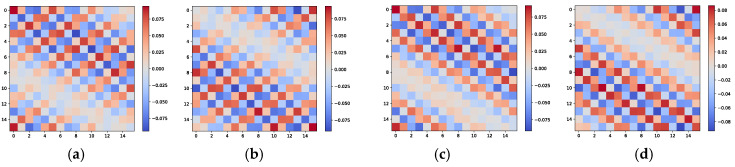
Image properties of the cross-covariance matrix ${\hat{R}}_{xz}$ for L-shaped array. (**a**) Real part of ${\hat{R}}_{xz}$ for $S_{1}=(-33{}^{\circ},-21{}^{\circ})$ and $S_{2}=(25{}^{\circ},42{}^{\circ})$. (**b**) Imaginary part of ${\hat{R}}_{xz}$ for $S_{1}=(-33{}^{\circ},-21{}^{\circ})$ and $S_{2}=(25{}^{\circ},42{}^{\circ})$. (**c**) Real part of ${\hat{R}}_{xz}$ for $S_{1}=(-33{}^{\circ},25{}^{\circ})$ and $S_{2}=(-21{}^{\circ},42{}^{\circ})$. (**d**) Imaginary part of ${\hat{R}}_{xz}$ for $S_{1}=(-33{}^{\circ},25{}^{\circ})$ and $S_{2}=(-21{}^{\circ},42{}^{\circ})$.

The graphical properties of the cross-covariance matrix ${\hat{R}}_{xz}$ can guide the network in associating θ with the correct φ. An example is presented below to demonstrate this. Let us consider an L-shaped array arranged as shown in Figure 1, where each uniformly spaced linear array includes 16 ideal array elements. Two independent and identically distributed signals, $S_{1}$ and $S_{2}$, are present in space, with their azimuth and elevation incident angles taking values in the sets θ∈(−33°,42°) and φ∈(−21°,25°), respectively. According to combinatorial principles, θ and φ have two possible combinations. The image characteristics of the cross-covariance matrix ${\hat{R}}_{xz}$ corresponding to these two signal arrangements are shown in Figure 2. To provide a more intuitive visualization of the angular relationships contained in the cross-covariance matrix, we adopt 16 array elements in this illustrative example. It is apparent that the image characteristics of the cross-covariance matrix ${\hat{R}}_{xz}$ for different signal combinations, while similar, exhibit noticeable texture differences, such as in the orientation of the patterns and the positions of larger elements. These differences can be detected by the neural network and incorporated into the parameter matching process.

As explained in the proposed method, we perform 2D-DOA estimation by using ${\hat{R}}_{xx}$, ${\hat{R}}_{zz}$, and ${\hat{R}}_{xz}$.

## 3. Proposed Method

**Figure 3:**
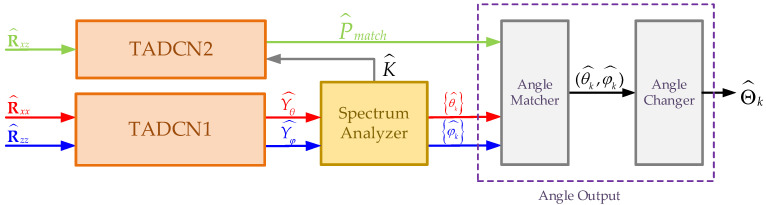
Proposed 2D-DOA estimation method.

For simplicity, we assume that $M_{z}=M_{x}=8$ and that the spacing of the adjacent elements of each subarray is λ/2. However, it is important to note that this assumption is made for convenience. The proposed algorithm establishes a mapping between the covariance matrix and DOA and can be applied to any arbitrary L-shaped array, including non-uniform arrays, as long as the covariance matrix can be computed. In this paper, we propose a new TADCN model which is used to train two neural networks: one for DOA estimation and the other for angle combination matching. This process can effectively reduce complexity and improve the accuracy of 2D-DOA estimation. Typically, 2D-DOA estimation requires the network to generate a 2D spatial spectrum. For example, the angle space in θ and φ is discretized, which results in the 2D spatial spectrum becoming a matrix of size $I_{\theta }\times I_{\varphi }$, where $I_{\theta }$ and $I_{\varphi }$ represent the grid sizes of the angles θ and φ, respectively. Such an extensive size of output is hard to deal with. Moreover, the complexity of peak identification within the network is approximately $O(I_{\theta }I_{\varphi })$. By using separate networks for angle estimation and matching, the overall complexity is reduced to $O(I_{\theta }+I_{\varphi })$. The proposed method consists of four consecutive steps, outlined as follows, with detailed descriptions of each step being provided in the subsequent sections:

1. The estimation covariance matrices ${\hat{R}}_{xx}$ and ${\hat{R}}_{zz}$ are used as the inputs of the angle estimation network (TADCN1), which outputs the pseudo-spectra $\hat{Y_{\theta }}$ and $\hat{Y_{\varphi }}$.
2. The estimation cross-covariance matrix ${\hat{R}}_{xz}$ of the received signals from the x-axis and z-axis subarrays is as the input of the angle matching network (TADCN2), which outputs the probabilities of different angle combinations generated by the possible values of all elements in the sets $\left\{\hat{\theta_{k}}\right\}$ and $\left\{\hat{\varphi_{k}}\right\}$.
3. By using the two pseudo-spectra ($\hat{Y_{\theta }}$, $\hat{Y_{\varphi }}$) from the angle estimation network, the spectrum analyzer module estimates the angle between the incident direction and the x-axis $\left\{\hat{\theta_{k}}\right\}$, the angle relative to the z-axis $\left\{\hat{\varphi_{k}}\right\}$, and the number of signals K.
4. Finally, the estimated $\left\{\hat{\theta_{k}}\right\}$, $\left\{\hat{\varphi_{k}}\right\}$, and angle combinations ${\hat{P}}_{match}$ from the angle matching network are fed into the angle output module to convert the estimated angles into a suitable form (the azimuth angle and elevation angle) for practical applications (see Figure 3).

### 3.1. The Architecture of the Proposed TADCN

As mentioned earlier, the architecture of the proposed method consists of two neural networks for estimation and matching, respectively, which are all based on the proposed TADCN model and different only after the flattening layer. Therefore, we take the angle estimation network (TADCN1) as an example to describe the framework in this section. The proposed TADCN model for DOA estimation is integrated by an innovative combination of triple attention mechanisms (TAMs) and deep convolutional networks (DCNs), which combines the advantages of convolutional layers and multi-dimensional feature interactions to accurately capture the angular characteristics of the signals in the space.

**Figure 4:**
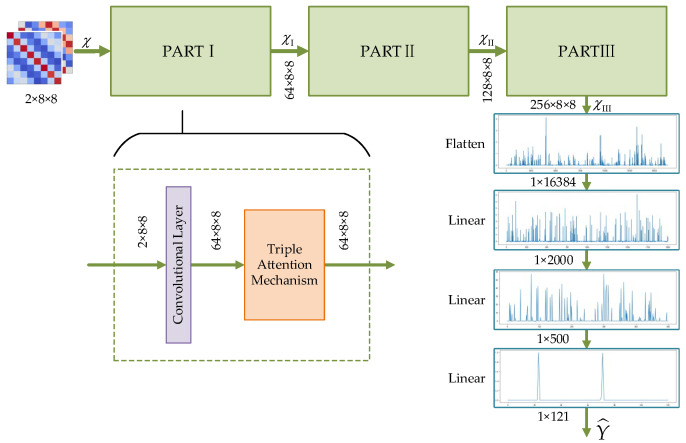
Proposed TADCN1 architecture.

The inputs to the TADCN1 model are the estimation covariance matrices ${\hat{R}}_{xx}$ and ${\hat{R}}_{zz}$, which are split into the real part and the imaginary part as two channels to facilitate network processing. The overall structure of the TADCN1 model is shown in Figure 4. There are three network architectures with the same structure, PARTI, PARTII, and PARTIII, to deal with the two channels including comprehensive signal feature information to progressively analyze and extract deeper angle information. As shown in Figure 4, PARTI consists of a convolutional layer and a TAM layer. After each convolutional layer, a TAM module is added to enhance feature representation. Each layer uses the Leaky ReLU activation function, which is similar to ReLU but outputs a small linear portion when the input is less than or equal to zero. This process is especially useful in deep networks to avoid the ‘dead neuron’ problem. Finally, the features extracted from PARTIII are flattened into a one-dimensional vector which is fed into fully connected layers to reduce the dimensionality gradually and convert the features into an estimated pseudo-spectrum.

### 3.2. Triple Attention Mechanism

The TAM can be adopted to capture the complex dependencies among the three dimensions of the input tensor: channel (C), height (H), and width (W). Although traditional channel attention mechanisms are computationally efficient and effective, they fail to capture spatial information within feature maps. They only compute scalar weights for each channel (e.g., via global average pooling), resulting in the spatial dimension typically being compressed into a single pixel per channel. In contrast to conventional attention mechanisms, the TAM is a nearly parameter-free attention mechanism that overcomes the limitations of independently computing channel and spatial attention in traditional methods by introducing the concept of cross-dimensional interaction. It captures the dependencies between the channel and spatial dimensions through three parallel branches and effectively compresses the channel dimension while preserving spatial information by using the Z-pool operation. This design not only enhances the ability of the model to integrate multi-dimensional information but also avoids the compression of spatial information in the input tensor. Therefore, the model we used can represent complex features to improve the estimate performance. The TAM, with its efficient and lightweight design, integrates multi-dimensional interactions between channels and spatial information without increasing the computational burden. This makes it possible to use the TAM to extract angular information from features in an efficient manner for DOA estimation tasks in complex signal environments.

The Z-pool operation is one of the key operations in the TAM. To facilitate subsequent lightweight computations while effectively preserving spatial information, Z-pool performs max pooling and average pooling along the zeroth dimension of the input tensor. Then, we concatenate the results along the zeroth dimension. This operation compresses the depth of the original tensor into two dimensions. Mathematically, the Z-pool operation can be expressed as

(10) $$Z-\mathrm{pool}(\chi )=[{\mathrm{MaxPool}}_{0d}(\chi ),{\mathrm{AvgPool}}_{0d}(\chi )]$$

where 0d denotes the zeroth dimension. For example, given a tensor $\chi \in {\mathbb{R}}^{C\times H\times W}$, the output tensor will have the shape (2×H×W).

Based on the previously defined operations, the TAM model we use to estimate DOAs consists of three independent attention pathways, each receiving the input tensor and outputting a tensor of the same shape. Every pathway focuses on different dimensional interactions and reconstructs refined feature representations through attention weights. Given an input tensor $\chi \in {\mathbb{R}}^{C\times H\times W}$ with *C* = 64, *H* = 8, and *W* = 8, the detailed process is sequentially defined as follows.

1. Channel–Height Branch

In this branch, the input tensor χ is rotated 90° anticlockwise along the height axis, denoted by $\chi^{1}$, which is used to reconstruct the relationship between the channel and height dimensions. After using the Z-pool operation to compress the width dimension, the tensor undergoes convolution and generates the attention weight $\omega_{1}$ multiplied by the rotated tensor $\chi^{1}$. Then, we rotate the multiplication clockwise to ensure that the output tensor has the same dimension as the input tensor, denoted by $\chi^{1^{*}}$.

2. Channel–Width Branch

Similarly, the input tensor χ is rotated $90^{{}^{\circ}}$ along the width axis in this branch, denoted by $\chi \chi^{2}$, which is used to model the dependency between the channel and width dimensions. After the Z-pool operation, the shape of the input tensor becomes (2×C×W) and then passes through a convolutional layer with sigmoid activation to generate the attention weight $\omega_{2}$, which is multiplied by $\chi^{2}$. Subsequently, the output of the multiplier is rotated clockwise to restore its original shape, denoted by $\chi^{2*}$.

3. Spatial Attention Branch

For the third branch, the Z-pool operation is directly applied to compress the channel dimension to 2, thereby generating attention weight $\omega_{3}$ along the spatial dimensions of height and width. The weight $\omega_{3}$ is directly multiplied by the input tensor $\chi^{3}$ to capture dependencies in the spatial dimensions, denoted by $\chi^{3^{*}}$.

The final attention-optimized tensor is obtained by averaging the outputs of the three branches:

(11) $$\chi_{I}=\frac{1}{3}\left(\chi^{1^{*}}+\chi^{2^{*}}+\chi^{3^{*}}\right)$$

**Figure 5:**
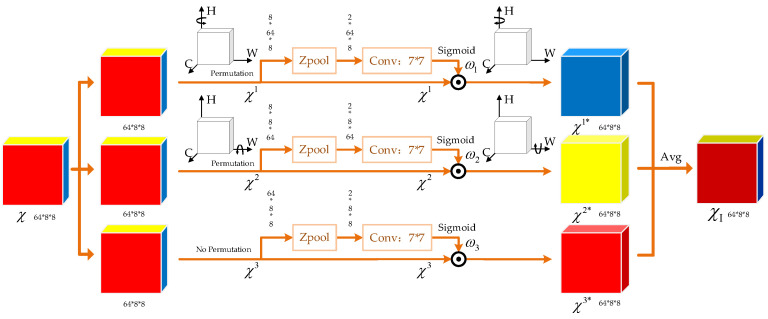
TAM architecture.

The resulting tensor $\chi_{I}$ captures the interdependencies across all three dimensions, enabling a more comprehensive feature representation. This TAM module that makes multi-dimensional interactions possible is essential to improving performance in DOA estimation tasks. The processing flow of the TAM after receiving $\hat{R}\in {\mathbb{R}}^{2\times 8\times 8}$ is shown in Figure 5.

### 3.3. Spectrum Analyzer

In the proposed method, we analyze the spatial spectrum from TADCN1 and employ an angle estimation technique based on linear interpolation to obtain precise estimation results. First, a copy of the original spatial spectrum $\hat{Y_{\theta }}$ is constructed as $\hat{Y_{\theta^{\prime }}}$, where the values at the indexes of the main peaks with K are set to zero. This operation is performed to suppress the influence of the main peaks on the search for the secondary peaks and to facilitate an accurate search for the position of the secondary peaks within a small range near the main peaks. The position of the secondary peak $j_{k}$ is determined by identifying the maximum value within the local region surrounding the main peak index $i_{k}$, specifically in the range from $i_{k}-1$ to $i_{k}+1$. Formula (12) gives the index of the secondary peak $j_{k}$:

(12) $$j_{k}=index\left(\max\left(\hat{Y_{\theta^{\prime }}}[i_{k}-1:i_{k}+1]\right)\right)+(i_{k}-1)$$

where $index\left(\max\left(\cdot \right)\right)$ denotes the index of the maximum value within a subinterval around the main peak $i_{k}$. Since the search range is localized relative to the main peak (i.e., within the interval $\left[i_{k}-1,i_{k}+1\right]$), the returned index corresponds to a local index. The adjustment $\left(i_{k}-1\right)$ is added to convert this into a position in the global spatial spectrum. Upon determining the indices of the main and secondary peaks, the corresponding amplitudes are recorded. The amplitude of the main peak is denoted by $A_{\mathrm{main}}=\hat{Y_{\theta }}[i_{k}]$, and the amplitude of the secondary peak is $A_{\sec\mathrm{ondary}}=\hat{Y_{\theta }}[j_{k}]$. By using these amplitudes, the contribution weights of each peak to the final angle estimate are calculated. Specifically, the contribution weights $\varpi_{1}$ and $\varpi_{2}$ for the main and secondary peaks are computed as follows:

(13) $$\varpi_{1}=\frac{A_{\mathrm{main}}}{A_{\mathrm{main}}+A_{\mathrm{secondary}}},\;\varpi_{2}=\frac{A_{\mathrm{secondary}}}{A_{\mathrm{main}}+A_{\mathrm{secondary}}}$$

where $\varpi_{1}$ and $\varpi_{2}$ represent the relative contributions of the main and secondary peaks to angle estimation. Given that the contributions of the main and secondary peaks are unequal, these weight coefficients effectively reflect the relative influence of each peak. The final angle estimate is obtained by computing the weighted average of the main and secondary peak indices. Precisely, the angle estimate $\hat{\theta_{k}}$ is calculated as follows:

(14) $$\hat{\theta_{k}}=\varpi_{1}\cdot i_{k}+\varpi_{2}\cdot j_{k}-60{}^{\circ}$$

The adjustment term −60° accounts for the offset in the angle range. Unlike traditional methods that rely solely on the main peak, the proposed spectrum analyzer reduces quantization errors by employing the weighted linear interpolation approach using both peaks. This operation will provide greater stability and accuracy, particularly in scenarios with peak overlap or significant noise interference.

### 3.4. Angle Output

The angle output module of the proposed method comprises two components: the angle matcher and the angle changer. The angle matcher is used to perform angle matching, and the angle changer converts the incident directions relative to the x-axis and z-axis into azimuth and elevation angles. The values $\hat{\theta_{k}}$ and $\hat{\varphi_{k}}$ derived from the spectrum analyzer is entered into the angle matching network, which outputs the most probable angle pair to provide the final 2D-DOA estimation. For k=2, the specific procedure is described in Algorithm 1.
**Algorithm 1: Angle matching process.****Inputs:** $\hat{\theta_{k}}$$,\;\hat{\varphi_{k}}$$,\;{\hat{P}}_{\mathrm{match}}$**Output:** ${\hat{C}}^{*}$$\mathrm{Use}\;\mathrm{spectrum}\;\mathrm{analyzer}\;\mathrm{to}\;\mathrm{obtain}\;\hat{\theta_{k}}$$\;\mathrm{and}\;\hat{\varphi_{k}}$Construct possible angle combinations: $\;\hat{C_{1}}=(\hat{\theta_{1}},\hat{\varphi_{1}},\hat{\theta_{2}},\hat{\varphi_{2}}),\;\hat{C_{2}}=(\hat{\theta_{1}},\hat{\varphi_{2}},\hat{\theta_{2}},\hat{\varphi_{1}})$Select the optimal combination:$\;{\hat{C}}^{*}=\underset{\hat{C}}{\arg\max}{\hat{P}}_{\mathrm{match}}(\hat{C})$$\mathrm{Return}\;{\hat{C}}^{*}$

In practical applications, however, to more accurately describe the DOA of the signal, it is necessary to define the azimuth $\vartheta_{k}$ as the angle between the incident direction of signal and its projection onto the z–y plane and the elevation $\phi_{k}$ as the angle between the incident direction of signal and its projection onto the x–y plane. This angle can be derived as follows:

(15) $$\cos\vartheta_{k}=\sin\theta_{k},k=1,\ldots ,K$$

(16) $$\cos\phi_{k}=\sin\varphi_{k},k=1,\ldots ,K$$

Upon completing these tasks, the angle changer outputs ${\hat{\Theta }}_{k}=\{\hat{\vartheta_{k}},\hat{\phi_{k}}\}$, facilitating the subsequent signal angle localization.

## 4. Data Generation and Network Training

The configuration of the antenna array and the TADCN model have been described in detail earlier. Here, we focus on data generation and network training. A diverse dataset is generated based on random source positions to train the angle estimation network and the angle matching network. For the subsequent simulation experiments, it is assumed that each sample contains two signal sources, i.e., K=2. The incident angles of the signals are randomly selected with the angle range defined as [−60°, 60°]. This angle range is chosen based on practical application scenarios. It effectively covers the spatial region observable by the L-shaped array and avoids the edge degradation commonly observed near detection boundaries. The grid scale is chosen as 1°, resulting in a total of 121 discrete angle values to balance computational efficiency and estimation accuracy. This generation scheme allows the training data to cover the entire spatial domain. The SNR is randomly selected within the range [−10,10] dB, and the number of snapshots is set to L=512. According to the sample design rules outlined above, 50,000 samples were generated randomly, and the dataset is split into training and test sets in a 0.8:0.2 ratio.

We employ the Adam optimizer with a fixed learning rate of 0.0012. At the end of each epoch, the loss trend is recorded to track learning progress and convergence. The training process of the TADCN model for angle estimation and matching is detailed in Algorithm 2, where ${\mathrm{model}}_{\theta }^{\left(0\right)}$, ${\mathrm{model}}_{\varphi }^{\left(0\right)}$, and ${\mathrm{model}}_{\mathrm{match}}^{\left(0\right)}$ denote the initial models for θ, φ, and angle matching processes before any optimization. The symbol L represents the loss function used to train the models in the estimation and matching tasks. The batch size during training is denoted by *B*. $N_{\mathrm{epochs},\;\mathrm{DOA}}$ and $N_{\mathrm{epochs},\;\mathrm{Match}}$ represent the number of epochs used for training the models related to DOA estimation and the angle matching process, respectively. The binary cross-entropy loss (BCELoss) function is used to quantify the discrepancy between predicted probabilities and true labels. The mathematical expression for BCELoss is given as

(17) $$\mathrm{BCELoss}(Y,\hat{Y})=-\frac{1}{N}\sum_{i=1}^{N}\left(Y_{i}\log(\hat{Y_{i}})+(1-Y_{i})\log(1-\hat{Y_{i}})\right)$$

where $Y_{i}$ is the true label, $\hat{Y_{i}}$ is the predicted value, and N is the number of samples.
**Algorithm 2: Training process for DOA estimation and angle matching.****Inputs** $\left({\hat{R}}_{xx},Y_{\theta }\right)$$,\;({\hat{R}}_{zz},Y_{\varphi })$$,\;({\hat{R}}_{xz},Y_{\mathrm{match}})$$,\;{\mathrm{model}}_{\theta }^{\left(0\right)}$$,\;{\mathrm{model}}_{\varphi }^{\left(0\right)}$$,\;{\mathrm{model}}_{\mathrm{match}}^{\left(0\right)}$, L, B$,\;N_{\mathrm{epochs},\;\mathrm{DOA}}$$,\;N_{\mathrm{epochs},\;\mathrm{Match}}$**Outputs** ${\mathrm{model}}_{\theta }^{*}$$,\;{\mathrm{model}}_{\varphi }^{*}$$,\;{\mathrm{model}}_{\mathrm{match}}^{*}$$\mathrm{for}\;n=1\;\mathrm{to}\;N_{\mathrm{epochs},\;\mathrm{DOA}}$ **do**$\mathrm{foreach}\;\mathrm{mini}-\mathrm{batch}\;({\hat{R}}_{xx}^{\left(i\right)},Y_{\theta }^{\left(i\right)})$B **do**${\hat{Y}}_{\theta }^{{\,}^{\left(i\right)}}\leftarrow {\mathrm{model}}_{\theta }^{{\,}^{\left(n-1\right)}}({\hat{R}}_{xx}^{\left(i\right)})$$L_{\theta }\leftarrow L({\hat{Y}}_{\theta }{\,}^{\left(i\right)},Y_{\theta }^{\left(i\right)})$$L_{\theta }.\mathrm{backward}()$Optimizer Adam.step() Optimizer Adam.zero_grad()$\mathrm{foreach}\;\mathrm{mini}-\mathrm{batch}\;({\hat{R}}_{zz}^{\left(i\right)},Y_{\varphi }^{\left(i\right)})$ of *size B* **do**${\hat{Y}}_{\varphi }^{{\,}^{\left(i\right)}}\leftarrow {\mathrm{model}}_{\varphi }^{{\,}^{\left(n-1\right)}}({\hat{R}}_{zz}^{\left(i\right)})$$L_{\varphi }\leftarrow L({\hat{Y}}_{\varphi }^{{\,}^{\left(i\right)}},Y_{\varphi }^{\left(i\right)})$$L_{\varphi }.\mathrm{backward}()$Optimizer Adam.step() Optimizer Adam.zero_grad()${\mathrm{model}}_{\theta }^{*}\leftarrow {\mathrm{model}}_{\theta }^{{\,}^{\left(N_{\mathrm{epochs},\;\mathrm{DOA}}\right)}}$${\mathrm{model}}_{\varphi }^{*}\leftarrow {\mathrm{model}}_{\varphi }^{{\,}^{\left(N_{\mathrm{epochs},\;\mathrm{DOA}}\right)}}$$\mathrm{for}\;n=1\;\mathrm{to}\;N_{\mathrm{epochs},\;\mathrm{Match}}$** do**$\mathrm{foreach}\;\mathrm{mini}-\mathrm{batch}\;({\hat{R}}_{xz}^{\left(i\right)},Y_{\mathrm{match}}^{\left(i\right)})$ of *size B* **do**${\hat{P}}_{\mathrm{match}}^{{\,}^{\left(i\right)}}\leftarrow {\mathrm{model}}_{\mathrm{match}}^{{\,}^{\left(n-1\right)}}({\hat{R}}_{xz}^{\left(i\right)})$$L_{\mathrm{match}}\leftarrow L({\hat{P}}_{\mathrm{match}}^{{\,}^{\left(i\right)}},Y_{\mathrm{match}}^{\left(i\right)})$$L_{\mathrm{match}}.\mathrm{backward}()$Optimizer Adam.step() Optimizer Adam.zero_grad()${\mathrm{model}}_{\mathrm{match}}^{*}\leftarrow {\mathrm{model}}_{\mathrm{match}}^{{\,}^{\left(N_{\mathrm{epochs},\;\mathrm{Match}}\right)}}$$\mathrm{Return}\;{\mathrm{model}}_{\theta }^{*}$$,\;{\mathrm{model}}_{\varphi }^{*}$$,\;{\mathrm{model}}_{\mathrm{match}}^{*}$

**Figure 6:**
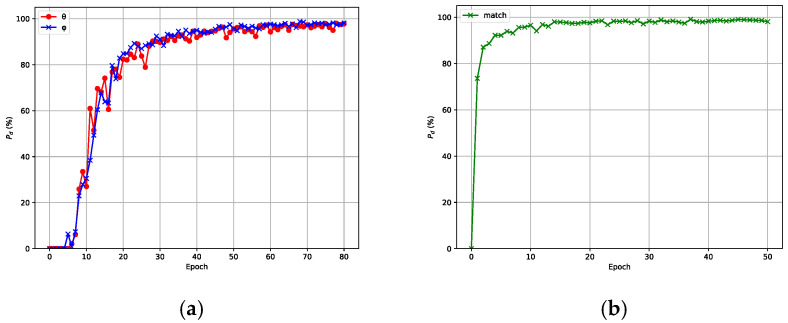
Model training and accuracy $P_{d}$. (**a**) TADCN1 training and accuracy $P_{d}$. (**b**) TADCN2 training and accuracy $P_{d}$.

By definition, the two signals are estimated accurately in a single trial if the absolute estimation error for both angles is no more than $1^{\circ }$. The accuracy $P_{d}$ is defined as

(18) $$P_{d}=\frac{n_{0}}{n}\times 100\%$$

where $n_{0}\leq n$, $n_{0}$ is the number of successful estimation samples, and n is the total number of test samples. The accuracy curve of the model with respect to the number of training iterations is shown in Figure 6, where (a) corresponds to the angle estimation network and (b) corresponds to the angle matching network. From Figure 6, we can see that the accuracy will not increase very much when the epoch number reaches a certain value. Considering the training cost and overall performance, the number of epochs for the angle estimation network was designed as 70, while the number of epochs for the angle matching network was set to 45. Table 1 shows the network structures of the angle estimation and matching networks trained under the aforementioned parameter settings and procedures.

## 5. Simulation Results

In this section, we present several experimental results for the proposed source localization scheme. We compare the performance of the proposed algorithm with that of other state-of-the-art DL-based approaches, such as the DNN, the DCN, and the RDCN. We also compare it with non-DL-based methods, including MUSIC, R-MUSIC, and ESPRIT.

### 5.1. DOA Estimation Performance in Scope of DOA Range

**Figure 7:**
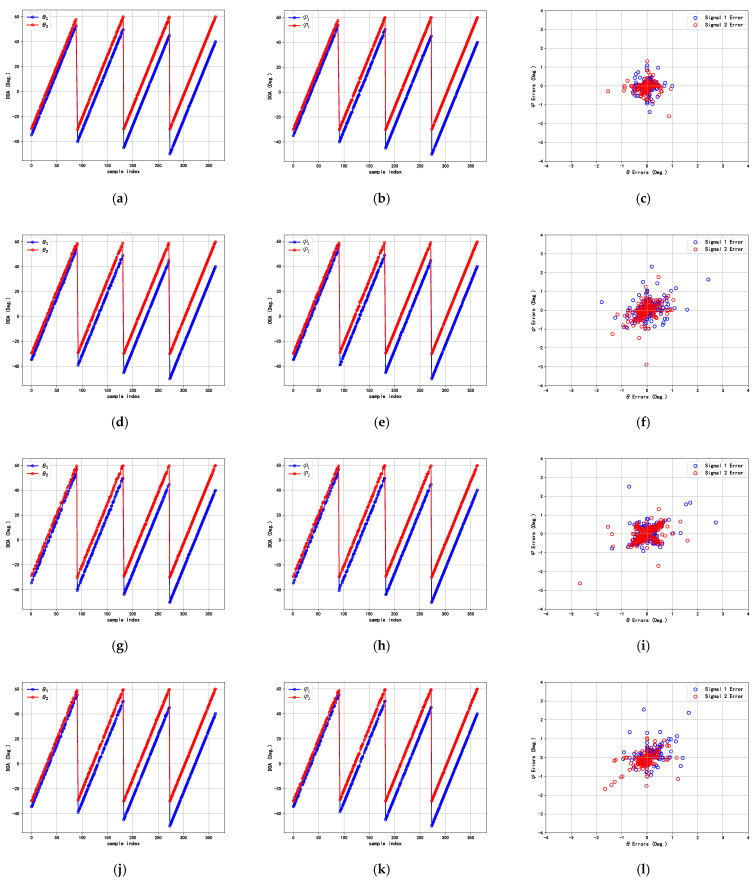
DOA estimation performance of different deep learning methods. (**a**) Proposed method for θ. (**b**) Proposed method for φ. (**c**) Estimation errors of proposed method. (**d**) DCN for θ. (**e**) DCN for φ. (**f**) Estimation errors of DCN. (**g**) DNN for θ. (**h**) DNN for φ. (**i**) Estimation errors of DNN. (**j**) RDCN for θ. (**k**) RDCN for φ. (**l**) Estimation errors of RDCN.

To assess the effectiveness of the proposed method, we design this experiment to estimate the azimuth and elevation angles, compared with the DCN, the DNN, and the RDCN. Here, we consider the scenario of two sources. The first incident angles, $\theta_{1}$ and $\varphi_{1}$, are set to vary between −30° and 60° with a step size of 1°. For the second incident angles, $\theta_{2}$ and $\varphi_{2}$, angular offsets, Δθ and Δφ, are introduced with values of −5°, −10°, −15°, and −20°. Simultaneously, the SNR is set to 20 dB, and the number of snapshots is set to 512 for sample generation. This linear interval variation is adopted to ensure that the experimental data covered a wide range of DOA angle combinations, providing strong support for the training and validation of the algorithm. Figure 7 presents the DOA estimation performance of the proposed method and the compared methods.

From Figure 7, it can be observed that the DCN, the DNN, and the RDCN have large DOA estimation errors for θ and φ, especially in smaller angular intervals (Δθ and Δφ), while the proposed method yields a lower estimation error, and the estimated trajectories closely matched the true signals for two signals, indicating that they have better DOA performance.

### 5.2. Network Performance

**Figure 8:**
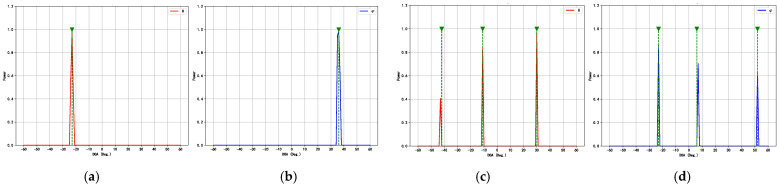
Network performance for varying numbers of signal sources. (**a**) θ=−23°. (**b**) φ=36°. (**c**) θ∈(−42°,−24°,31°). (**d**) φ∈(−11°,5°,52°).

In this section, we verify the generalization performance of the proposed network model, which is trained by using samples generated from two signal sources. The settings for the SNR and the number of snapshots remain the same as those in 6.1. The proposed network model is capable of performing DOA estimation under varying numbers of incident signal sources (K). Figure 8a,b display the estimation results for K=1, with incident angles of (−23°,36°). Figure 8c,d show the estimation results for K=3, with incident angles of (−42°,5°), (−24°,−11°), and (31°,52°). These results demonstrate that the proposed network model possesses strong generalization capabilities, enabling it to effectively handle more complex signal scenarios.

### 5.3. Correlation Test

**Figure 9:**
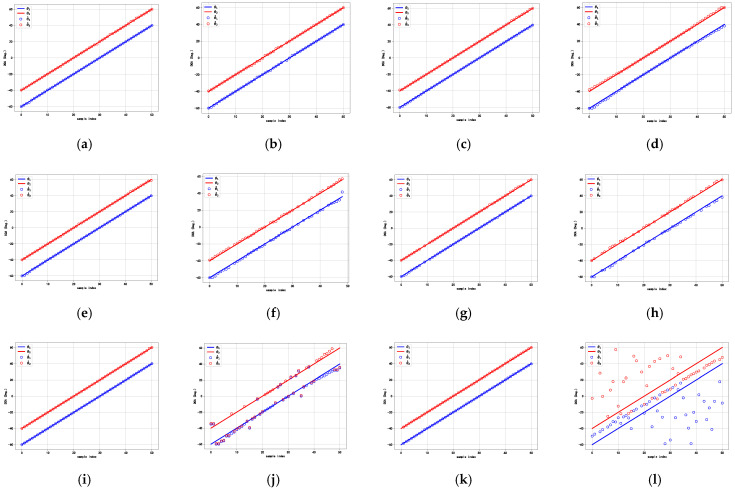
Estimation performance of various methods for signal sources with varying correlations. (**a**) Proposed method for ρ=0.1. (**b**) Proposed method for ρ=0.99. (**c**) DNN for ρ=0.1. (**d**) DNN for ρ=0.99. (**e**) DCN for ρ=0.1. (**f**) DCN for ρ=0.99. (**g**) RDCN for ρ=0.1. (**h**) RDCN for ρ=0.99. (**i**) MUSIC for ρ=0.1. (**j**) MUSIC for ρ=0.99. (**k**) ESPRIT for ρ=0.1. (**l**) ESPRIT for ρ=0.99.

In this section, we generate two sets of testing data under correlation coefficients ρ=0.1 and ρ=0.99 for angle directions by using the array along the *x*-axis as an example. The angle of the first signal varies from −60° to 40° with a step size of 2°, and the angle of the second signal has an angular separation of Δθ=20° relative to the first signal. Additionally, both signals are set with an SNR of 0 dB, and the number of snapshots is set to 512. Figure 9 illustrates the estimation results of the proposed method compared with the existing state-of-the-art approaches, under the conditions of correlation coefficients of 0.1 and 0.99, respectively. It can be observed that the proposed method exhibits highly consistent estimation performance in both incoherent and coherent environments and outperforms other existing methods in signal source coherence. The superior performance is due to the fact that the proposed method introduces the TAM into the convolutional layers to better differentiate and reinforce features from different signal sources when handling highly correlated sources. The performance of the DNN is relatively good in both coherent and incoherent environments, due to the simplicity of its model to reduce the risk of overfitting. In contrast, the DCN and the RDCN perform poorly in coherent environments, particularly near the boundary of the angular range. For non-deep learning methods, despite significant angular separation between DOAs, the performance of the MUSIC and ESPRIT methods severely degrades when the two signals are highly correlated.

### 5.4. Statistical Characteristics

In this section, the statistical performance of the proposed method is discussed, comparing it with other methods by using the root mean square error (RMSE) and accuracy $P_{d}$ previously defined as evaluation metrics. The RMSE for 2D-DOA estimation is computed as follows:

(19) $$\mathrm{RMSE}=\frac{1}{\sqrt{2PK}}\sqrt{\sum_{p=1}^{P}\sum_{k=1}^{K}\left[{\left(\theta_{kp}-{\hat{\theta }}_{kp}\right)}^{2}+{\left(\varphi_{kp}-{\hat{\varphi }}_{kp}\right)}^{2}\right]}$$

where ${\hat{\theta }}_{kp}$ and ${\hat{\varphi }}_{kp}$ are the estimated angles for the k-th signal in the p-th Monte Carlo trial, while $\theta_{kp}$ and $\varphi_{kp}$ represent the corresponding truth angles. P and K denote the number of Monte Carlo trials and signals, respectively. The DOA estimation performance is evaluated under varying SNR conditions. Specifically, two equal-power signals are simultaneously incident on the L-array. The incident angle of signal 1, $\left(\theta_{1},\varphi_{1}\right)$ is set to (−20°+ξ,10°+ξ), and the incident angle of signal 2, $\left(\theta_{2},\varphi_{2}\right)$ is set to (20°+ξ,30°+ξ), where ξ is randomly selected from the range [−0.5°,0.5°]. Monte Carlo simulations (600 trials) are conducted for SNR values ranging from −10 dB to 10 dB, with the number of snapshots L=512.

**Figure 10:**
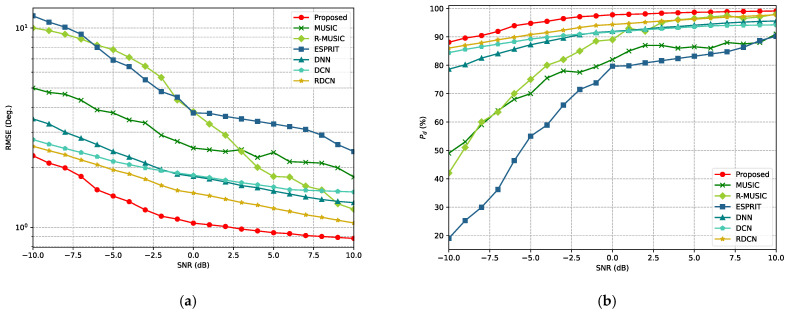
RMSE and $P_{d}$ curves of various methods for different SNRs. (**a**) RMSE curves for different SNRs. (**b**) $P_{d}$ curves for different SNRs.

As illustrated in Figure 10a, the curves of the RMSE for all methods generally decrease as the SNR increases. However, the proposed method consistently achieves a lower RMSE across the entire SNR range. In the low-SNR region (−10 dB to 0 dB), the RMSE curve of the proposed method is significantly lower than that of traditional methods (e.g., MUSIC and ESPRIT) and other deep learning methods (e.g., DNN, DCN, and RDCN). This demonstrates the effectiveness of the proposed method in DOA estimation under high-noise or weak-signal conditions, showcasing its robustness and stability. Similarly, in Figure 10b, as the SNR increases, the detection probability for each method improves. The proposed method maintains a high detection probability across all SNR levels, with accuracy close to 90% even at −10 dB. In contrast, other methods generally perform poorly under low-SNR conditions, especially traditional methods like MUSIC and ESPRIT.

**Figure 11:**
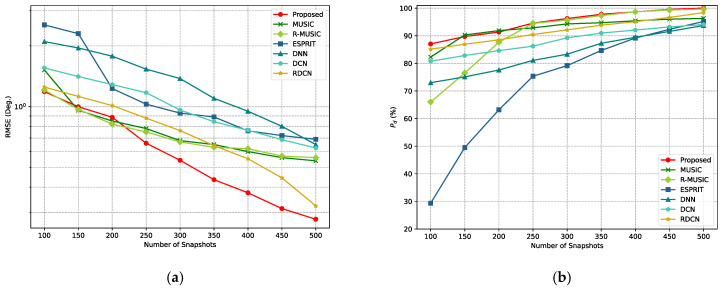
RMSE and $P_{d}$ curves for different snapshot numbers for various methods. (**a**) RMSE curves for different snapshot numbers. (**b**) $P_{d}$curves for different snapshot numbers.

Subsequently, the performance of the proposed method was evaluated under different numbers of snapshots, with the signal angles fixed at (−21°,13°) and (7°,34°). The SNR was set to 10 dB. For each snapshot configuration, 600 Monte Carlo experiments were conducted. The results are shown in Figure 11. From Figure 11a, it can be observed that the curves of the RMSE for all algorithms decrease when the number of snapshots increases. However, the RMSE curve for the proposed method remains significantly lower than that of the other methods under all snapshot conditions, and its RMSE curve decreases more rapidly with the increase in the number of snapshots. In Figure 11b, the detection accuracy for all algorithms improves as the number of snapshots increases. However, the detection probability for the proposed method exceeds 90% with only 200 to 300 snapshots, far outperforming other methods.

**Figure 12:**
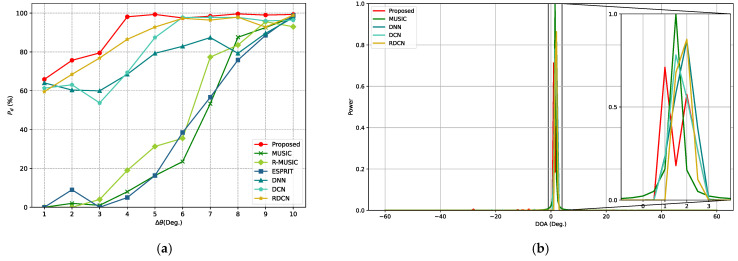
Resolution performance comparison of different methods. (**a**) $P_{d}$ curves for different angular separations Δθ. (**b**) Spatial spectra of different methods for two sources with closely spaced angles.

Additionally, we conducted experiments to evaluate the resolution accuracy of the proposed method and compare its ability with other models to resolve adjacent signal sources. Specifically, the first incident angle $\theta_{1}$ was fixed at 0°, while the second incident angle $\theta_{2}$ varied with an angular offset Δθ ranging from 1° to 10° in 1° increments. The SNR was set to 10 dB, and the number of snapshots was fixed at 512 for each sample. For every angular separation Δθ, 600 Monte Carlo trials were conducted, and the detection accuracy $P_{d}$ of each method was computed, as shown in Figure 12a. The results clearly demonstrate that the proposed method significantly outperforms both traditional approaches and other deep learning models in terms of resolution capability. Notably, with an angular separation as small as 4°, the proposed model maintains high accuracy, indicating its superior robustness and feature extraction performance in scenarios involving closely spaced sources.

Super-resolution capability refers to the ability of a method to resolve sources separated by angular distances smaller than the Rayleigh limit, which is typically constrained by factors such as the array aperture and configuration. Notably, the resolution of spatial spectra generated by deep learning models is influenced by the angular grid size used during training and inference. A coarse grid may limit the ability of models to distinguish closely spaced sources. To further evaluate the super-resolution capability of the proposed method, we reduced the angular grid size to 0.5° and conducted an experiment involving two sources with closely spaced incident angles at 1.37° and 2.15°. The spatial spectra of all applicable methods are shown in Figure 12b. It shows that the proposed method still produces two clearly distinguishable and sharp peaks in the spatial spectrum, reflecting its strong super-resolution capability. In contrast, the spectra produced by other deep learning models and the conventional MUSIC algorithm produce only one peak, failing to separate the two signals. It should be noted that R-MUSIC and ESPRIT are not spatial spectrum-based algorithms and are therefore excluded from Figure 12b.

## 6. Conclusions

In this paper, we propose a novel 2D-DOA estimation algorithm using an L-shaped array. Due to the innovative network architecture and optimization strategy, the proposed algorithm can achieve significant improvements in both the accuracy of angle estimation and efficiency. These have been demonstrated by theoretical analysis and the simulation experiment. The proposed TADCN model (used for angle estimation and pairing) is able to capture the interdependencies among the channel, height, and width dimensions of the signals by introducing the TAM to construct a DCN network. It exhibits superior accuracy and excellent error control, particularly in multi-signals where the signal angles are closely spaced. Additionally, this algorithm can be directly applied to the correlated signals without any additional operations. In contrast to traditional model-based DOA estimation methods, such as MUSIC and ESPRIT, the proposed method does not require intricate mathematical models or prior knowledge. Compared with current deep learning methods (e.g., DNN, DCN, and RDCN), it has a superior performance in a range of SNR and snapshot conditions. In future work, we plan to extend the proposed method to MIMO systems and enhance its robustness under challenging conditions such as low SNR, limited snapshots, and multiple sources by incorporating more diverse training scenarios.

## Figures and Tables

**Table 1:** The detailed architecture and parameters of DOA EstimationNet and AngleMatchingNet.

DOA EstimationNet		AngleMatchingNet	
Layer	Configuration	Layer	Configuration
Input	Input Shape: (*B*, 2, H, W)	Input	Input Shape: (*B*, 2, H, W)
Conv Layer 1	Conv2d(2, 64, 3 × 3)	Conv Layer 1	Conv2d(2, 16, 3 × 3)
	BatchNorm2d(64) + LeakyReLU		BatchNorm2d(16) + LeakyReLU
TripletAttention	C, H, W	TripletAttention	C, H, W
Conv Layer 2	Conv2d(64, 128, 3 × 3)	Pooling Layer 1	MaxPool2d(2 × 2)
	BatchNorm2d(128) + LeakyReLU		Conv2d(16, 32, 3×3) + BatchNorm2d(32) + LeakyReLU
TripletAttention	C, H, W	TripletAttention	C, H, W
Conv Layer 3	Conv2d(128, 256, 3 × 3)	Pooling Layer 2	MaxPool2d(2 × 2)
	BatchNorm2d(256) + LeakyReLU		Conv2d(32, 64, 3 × 3)+BatchNorm2d(64) + LeakyReLU
TripletAttention	C, H, W	TripletAttention	C, H, W
Flatten	Output Shape: (*B*, 256 × H × W)	Pooling Layer 3	MaxPool2d(2 × 2)
FC Layer 1	Linear(256 × H × W, 2000) + Dropout (*p* = 0.2)	Flatten	Output Shape: (*B*, 256)
	LeakyReLU		
FC Layer 2	Linear(2000, 500) + LeakyReLU	FC Layer 2	Linear(256, 128) + LeakyReLU
FC Layer 3	Linear(500, 121) + Sigmoid	FC Layer 3	Linear(128, 2) + Softmax

## Data Availability

The original contributions presented in the study are included in the article, and further inquiries can be directed to the corresponding author.
